# Sexual Dysfunction and Associated Anxiety and Depression in Female Hemodialysis Patients: A Cross-Sectional Study at Karachi Institute of Kidney Diseases

**DOI:** 10.7759/cureus.10148

**Published:** 2020-08-31

**Authors:** Sadia Yaqoob, Mahjabeen Yaseen, Hassan Abdullah, Furqan Ahmad Jarullah, Uzzam Ahmed Khawaja

**Affiliations:** 1 Medicine, Jinnah Sindh Medical University, Karachi, PAK; 2 Nephrology, Fazaia Ruth Pfau Medical College, Karachi, PAK; 3 Neurology, University of Alabama, Birmingham, USA; 4 Medicine, Nishtar Medical University, Multan, PAK

**Keywords:** sexual dysfunction, hemodialysis, anxiety, depression

## Abstract

Objectives

Hemodialysis patients have to combat certain negative effects such as sexual dysfunction, depression, and anxiety. This study aimed to measure the sexual function and identify the relationship between sexual dysfunction, depression, and anxiety in females undergoing hemodialysis.

Methods

The descriptive, cross-sectional study was conducted at a dialysis unit in November 2019. Forty-eight females were enrolled in the study. Participants were interviewed for sociodemographic, clinical, and biochemical parameters. Sexual function was assessed through the Female Sexual Function Index (FSFI) while depression and anxiety scores were calculated using the hospital anxiety and depression scale (HADS).

Results

In this study, the mean age of patients was 44.60 ± 10.27 years. Median sexual function scores were low across all domains. A maximum possible score of 3.4 was calculated for the satisfaction domain while the minimum score calculated was 0 for arousal, lubrication, and orgasm. 14.6% and 45.8% were suffering from borderline abnormal and abnormal depression, respectively. However, 33.3% and 31.3% had borderline abnormal and abnormal anxiety, respectively. Pearson’s correlation showed a significant negative correlation between age and desire domain (r = -0.343; p < 0.05) and demonstrated that arousal, lubrication, orgasm, satisfaction, and pain domains were associated with borderline abnormal depression. None of the sexual domains were correlated with anxiety.

Conclusions

Sexual dysfunction, depression, and anxiety are highly prevalent in hemodialysis patients. In this study, borderline abnormal depression was independently linked to sexual dysfunction excluding sexual desire. Therefore, healthcare teams should keep up with the progress of their patients and evaluate for psychosexual health so that they can be timely managed.

## Introduction

End-stage renal disease (ESRD) refers to chronic kidney disease that is so severe in which death is likely without renal replacement therapy (dialysis and renal transplantation) [1]. Currently, hemodialysis is trending since patients are falling short of organs to transplant due to the escalating global burden of ESRD [2]. Hemodialysis remains the treatment of choice also because it helps to optimize patients' functional status and increase life expectancy. However, those undergoing dialysis needs to cope up with certain negative effects including loss of libido, anxiety, and depression [3,4].

Sexual dysfunction refers to a lack of sexual interest or willingness [5]. The worldwide incidence of sexual dysfunction in female hemodialysis patients is reported to be 25-64% [6]. Common symptoms associated with female sexual dysfunction include low sexual desire, diminished vaginal lubrication, pain and discomfort upon intercourse, minimal sense of arousal, and difficulty in achieving orgasm [7]. However, only a small percentage of women seek medical attention since they are hesitant or embarrassed to discuss it.

Depression and anxiety are amongst the most prevalent psychological drawbacks of hemodialysis [8]. Nationwide, the estimated prevalence rates of depression and anxiety in 2015 were 4.4% and 3.6%, respectively, per WHO, which incremented by 18% over the period 2005-2015 [9].

Depression is a mood disorder featured by sadness, hopelessness, and loss of interest or pleasure in daily activities. Greater severity of depressive symptoms in dialysis patients is associated with higher risks for hospitalization, economic crisis, and death. Despondency becomes more pronounced when the patient gradually fails to adhere to the prescribed dialysis regimen, diet, and/or medications [10]. On a different note, the American Psychological Association defines anxiety as an emotion characterized by feelings of tension, worried thoughts, and physical changes like increased blood pressure [11]. After each dialysis episode, anxiety hovers over, culminating in tiredness (71%), itching (55%), appetite dysregulation (49%), pain (47%), insomnia (44%), and constant agitation (38%) [12].

Whilst dialysis units continue to expand all over Karachi, fewer studies have been concentrated here. However, rising rates of ESRD and its related morbidity and mortality have prompted us to evaluate the common issues faced by female hemodialysis patients. Can females under hemodialysis still enjoy sexual activity? Are depression and anxiety correlated with sexual dysfunction? Such questions stirred us to conduct our study where we aim not only to tie-up the links between sexual dysfunction, depression, and anxiety, but also gauge sexual functioning using the Female Sexual Function Index (FSFI).

## Materials and methods

Study setting

This study was conducted in a tertiary hemodialysis unit, Karachi Institute of Kidney disease, located in the F.B. Area of Karachi.

Subjects, sample size, and sampling technique

All females receiving hemodialysis during different work shifts on weekdays during November 2019 were approached. After having met the inclusion criteria, they were included in the study.

Study design

The research approach employed a descriptive, cross-sectional study to investigate sexual dysfunction and associated anxiety and depression in female hemodialysis patients.

Data collection tools

The objectives of the study were explained to each participant, and after obtaining the consent, subjects were interviewed as per the questions listed in the questionnaire. The questionnaire comprised of three sections: sociodemographic, clinical, and biochemical parameters constituted the first section which included age, number of children, weight, height, BMI, level of education, occupation, menstrual status, residence, cause of renal failure, duration of kidney disease, duration of hemodialysis, number of dialysis per week, a shift of dialysis, duration of each session of dialysis, any other comorbid, Hb levels, erythropoietin, Venofer injections, calcium, phosphorous, albumin, and parathyroid hormone levels.

The second section included the 19-item self-report questionnaire to measure sexual functioning in women - FSFI [13]. Over the past four weeks, six domains such as desire, arousal, lubrication, orgasm, satisfaction, and pain, each having a maximum score of 6, were assessed to construe the presence of sexual dysfunction if scores equal to or below 26.55 [14].

The third section encompassed the hospital anxiety and depression scale (HADS) questionnaire which is used to extrapolate the degree of depression and anxiety amongst subjects. HADS is a self-administered questionnaire consisting of seven-depression-related and seven-anxiety-related items [15]. For each variable, a score of 0-7 is considered normal; 8-10 is a borderline case, while 11-21 is considered an abnormal case.

Inclusion criteria

Patients included in the study were aged between 18 and 65 years, living with their husbands for the past one month and had complete medical records.

Exclusion criteria

Those excluded from the study were unmarried women or widows, women with a major psychiatric disorder, and those who declined to participate in the study.

Method of data analysis

Descriptive statistics were generated, based on frequencies for categorical data, or means ± standard deviations (SDs) for quantitative data. Pearson's correlation analysis was used to compare categorical variables. A P-value of <0.05 was considered statistically significant. All data were analyzed using SPSS Software 25.0 (SPSS, Inc., Chicago, USA).

Ethical consideration

Ethics approval was obtained from the Ethical Board Review while permission was taken from the institute. Consents and permission to access medical records were taken from all the participants and they were ensured that confidentiality will be granted to them.

## Results

After having met the inclusion criteria, 48 females volunteered to take part in the study. Table 1 exemplifies the demographics and clinical parameters of patients enrolled in the study.

**Table 1:** Demographics and clinical parameters of patients

Variable	Study samples (n = 48)
Patients demographics	
Age (years)	28-64
BMI (kg/m^2^) mean ± SD	22.38 ± 6.47
Education (n, %)	
Illiterate	11 (22.9)
Primary	4 (8.3)
Secondary	24 (50.0)
University education	9 (18.8)
Occupation (n, %)	
Working	2 (4.2)
Housewives	46 (95.8)
Menstrual status (n, %)	
Postmenopausal	29 (60.4)
With menstrual cycle	19 (39.6)
Residence (n, %)	
Urban	47 (97.9)
Rural	1 (2.1)
Duration of hemodialysis (months)	32.31 ± 40.61
Cause of renal failure (n, %)	
Diabetes mellitus	9 (18.8)
Hypertension	39 (81.3)
Glomerulonephritis	0
Renal stone disease	3 (6.3)
Urinary Infections	3 (6.3)
Cystic kidney disease	0
Unknown cause	2 (4.2)
Other diseases (n, %)	
Hepatitis B	1 (2.1)
Hepatitis C	6 (12.5)
Cardiovascular diseases	7 (14.6)
Shift (n, %)	
Morning	20 (41.7)
Afternoon	16 (33.3)
Evening	12 (25.0)
Patient clinical characteristics	
Hb (g/l)	9.72 ± 1.82
Calcium (mmol/l)	8.96 ± 1.31
Phosphorous (mmol/l)	5.87 ± 1.50
Albumin (g/l)	3.55 ± 0.74

Their mean age was 44.60 ± 10.27 while the average duration of hemodialysis was 32.31 ± 40.61 months. Most of the patients were postmenopausal (60.4%) and working as housewives (95.8%). The majority viewed that the leading causes of renal failure were diabetes (18.8%) and hypertension (81.3%).

As for the arousal, lubrication, orgasm, satisfaction, and pain domains, 33 patients (68.8%) had no sexual activity. Only one patient (2.1%) accounted for very low or no sexual arousal, two (4.2%) of them found it extremely difficult or impossible to lubricate, four patients (8.3%) had difficulty in reaching orgasm, none were very dissatisfied with their overall sex life, and only one patient (2.1%) reported to have high pain during sexual activity. Median scores enlisted in Table 2 were: desire (1.2), arousal (0), lubrication (0), orgasm (0), satisfaction (3.4), pain (0), and a total score (5.2).

**Table 2:** The score distribution of each sexual function domain

Domains	Mean ± SD	Median	25^th^ percentile	75^th^ percentile
Desire	1.925 ± 1.0052	1.2	1.2	2.85
Arousal	1.175 ± 1.8749	0	0	3
Lubrication	1.494 ± 2.3683	0	0	3.75
Orgasm	1.467 ± 2.2812	0	0	3.9
Satisfaction	3.658 ± 1.6357	3.4	2.4	5.4
Pain	1.233 ± 2.0477	0	0	2
Total score	10.702 ± 9.8308	5.2	4.25	20.075

Patients' HADS scores of anxiety and depression ranged from 0 to 21. Of all the patients assessed for depression, 19 (39.6%) were normal, 7 (14.6%) were borderline abnormal, and 22 (45.8%) were abnormal. However, the severity of anxiety differed a bit with 17 patients (35.4%) being normal, 16 (33.3%) with the borderline abnormal case, and 15 (31.3%) of being abnormal variety (Table 3).

**Table 3:** Severity of depression and anxiety in patients undergoing hemodialysis

Variables	Normal, n (%)	Borderline abnormal, n (%)	Abnormal, n (%)
Depression	19 (39.6)	7 (14.6)	22 (45.8)
Anxiety	17 (35.4)	16 (33.3)	15 (31.3)

Pearson's correlation was run to measure the association of age, depression, and anxiety with various sexual domains (Table 4).

**Table 4:** Pearson’s correlation coefficient results of age, depression, and anxiety with sexual function domains in hemodialysis *Correlation is significant at the 0.05 level (two-tailed).

		Desire score	Arousal score	Lubrication score	Orgasm score	Satisfaction score	Pain score
Age		R = -0.343^*^	R= -0.095	R= -0.089	R = -0.077	R = -0.071	R = -0.149
		P = 0.017	P = 0.521	P = 0.550	P = 0.601	P = 0.631	P = 0.311
Depression							
Normal		R = -0.287	R = -0.241	R = -0.061	R = -0.067	R = 0.011	R = -0.087
		P = 0.233	P = 0.323	P = 0.805	P = 0.785	P = 0.964	P = 0.723
Borderline abnormal		R = -0.298	R = -0.798^*^	R = -0.871^*^	R = -0.839^*^	R = -0.781^*^	R = -0.757^*^
		P = 0.516	P = 0.031	P = 0.011	P = 0.018	P = 0.038	P = 0.049
Abnormal		R = -0.262	R = -0.059	R = -0.248	R = -0.174	R = -0.068	R = -0.064
		P = 0.238	P = 0.794	P = 0.267	P = 0.439	P = 0.765	P = 0.778
Anxiety							
Normal		R = 0.259	R = -0.337	R = -0.227	R = -0.263	R = -0.360	R = -0.360
		P = 0.315	P = 0.187	P = 0.381	P = 0.307	P = 0.156	P = 0.156
Borderline abnormal		R = 0.154	R = -0.118	R = -0.070	R = -0.041	R = 0.021	R = -0.109
		P = 0.569	P = 0.665	P = 0.797	P = 0.880	P = 0.939	P = 0.689
Abnormal		R = -0.466	R = -0.359	R = -0.371	R = -0.372	R = -0.248	R = -0.360
		P = 0.080	P = 0.189	P = 0.173	P = 0.172	P = 0.373	P = 0.188

The results revealed a significant negative correlation between age and desire domain (r = -0.343; p < 0.05). The results also showed a negative relationship between arousal, lubrication, orgasm, satisfaction, and pain domain with borderline abnormal depression which were considered significant. Conversely, sexual domains were not significantly correlated with anxiety.

## Discussion

Even though the renal replacement therapy is suggestive of upgrading the quality of life of those suffering from ESRD, it has seriously hampered the psychosexual life of an individual [16].

Sexual dysfunction is multi-faceted and includes increasing age, menopausal status, depression, and comorbidities like diabetes and hypertension as its major predictors. Literature also shows a discrepancy in the relationship between older age and depression with sexual functioning [17,18]. In particular, the results of this study manifested that age had a significant negative correlation with sexual desire, but not with arousal, lubrication, orgasm, satisfaction, or pain. This implies that elderly women experienced a gradual decrease in libido as part of the aging phenomenon than merely due to the disease since the remaining sexual domains were not affected.

Several types of research are inclined towards assessing the degree of depression among hemodialysis patients, while sexual health remained under-recognized [19]. This study was improvised to evaluate the degree of sexual dysfunction in addition to depression and anxiety and find the association between them.

Sexuality was evaluated using a 19-item FSFI over the past four weeks in all six domains: sexual desire, arousal, lubrication, orgasm, satisfaction, and pain. Of all the domains, sexual desire was affected a great deal while none of them reported high levels of dissatisfaction concerning their sexual life. These findings implicit that sexual satisfaction has not just been boosted by increased sexual desire; additive analysis is required to evaluate biological, social, and other parameters influencing women's sexual satisfaction. Similar results were also seen in a study conducted by Saglimbene et al. [20] and Weisbord [21]. On account of sexual inactivity, median scores were nil across arousal, orgasm, lubrication, and pain subscales of FSFI, whereas the satisfaction score was still raised. This corroborates with the findings of a study conducted by Nasrallah et al. [22].

As the disease progresses, depression and/or anxiety sneak up and it not only damages an individual's self-esteem but also ruins sexual life [23]. Guven et al. found out that both anxiety and depression were significantly related to sexual dysfunction [24]. Contrary to the results from Esen et al. [5], the present study construed a significant negative correlation between borderline abnormal depression and all sexual domains except sexual desire; meaning borderline abnormal depression scores led to impaired sexual functioning but did not alter libido. Previous studies have failed to correlate the degree of depression with each subset of FSFI. However, anxiety and sexual dysfunction were not inter-related in the current study.

This study was limited in several aspects. First, the sample size was too small. Second, the results of a single-center approach were not sufficient to generalize the findings to the whole population. Third, other parameters impacting sexual life like sociocultural, hormonal, neurovascular, and medical effects were not looked upon. Hence, further evaluation can help to improve substantial findings.

## Conclusions

Patients undergoing hemodialysis not only have to battle the disease but also with subsequent complications arising from it. Depression and anxiety add up to the ordeal. In this study, borderline abnormal depression was independently linked to sexual dysfunction excluding sexual desire. The plan should be outlined to deal with the upcoming challenges, particularly those concerning mental and sexual health. Healthcare teams should keep up with the progress of their patients and evaluate such issues if suspicion arises. Patients should also be encouraged to discuss their health issues frankly with their doctors so that they can be timely evaluated and managed.
